# Supplementary material for: Volumetric and structural connectivity abnormalities co-localise in TLE
Source: Neuroimage Clin. 2022 Jul 9;35:103105. doi: 10.1016/j.nicl.2022.103105 (PMC9421455; doi:10.1016/j.nicl.2022.103105)
Supplement: Supplementary data 1 [file mmc1.docx]

Supplementary material:

Volumetric and structural connectivity abnormalities co-localise in TLE

Jonathan J. Horsley${}^{1}$, Gabrielle M. Schroeder${}^{1}$,
Rhys H. Thomas${}^{2}$, Jane de Tisi${}^{3}$, Sjoerd B. Vos${}^{3,4,5}$, Gavin P. Winston${}^{3,6}$,
John S. Duncan${}^{3}$, Yujiang Wang${}^{1,2,3}$ and Peter N. Taylor${}^{*1,2,3}$

**Supplementary Contents**

**Supplementary Methods 1 - Colocalisation**

**Supplementary Methods 2 - Different Analyses**

**Supplementary Analysis 1 - Repeatability Across Parcellation Schemes: Connections Between Abnormal Volumes**

**Supplementary Analysis 2 - Repeatability Across Parcellation Schemes: Volumes Joined by Abnormal Connections**

**Supplementary Analysis 3 - Repeatability Across Thresholds: Hierarchical Models**

**Supplementary Analysis 4 - Repeatability Across Thresholds: Colocalisation**

**Supplementary Analysis 5 - Hierarchical Models without Ipsilateral Temporal and Subcortical Regions**

**Supplementary Analysis 6 - Hierarchical Models without Ipsilateral Temporal and Subcortical Regions by Surgical Outcome Status**

**Supplementary Analysis 7 - Repeatability Using Axial Diffusivity (AD) and Radial Diffusivity**

**Supplementary Analysis 8 - Regions and Associated Lobe Table**

**Supplementary Methods 1 - Co-localisation**

A limitation of group-level analysis is that this does not provide information at an individual patient level. Consider the scenario illustrated below in Figure S1 in which 50% of a patient cohort have volume, but not connectivity, abnormalities in ipsilateral temporal regions (subpanel a). If the other 50% of patients have connectivity, but not volume, abnormalities in ipsilateral temporal regions (subpanel b) then, at a group level, the abnormalities would co-localise. However, in that example, not a single patient exhibits *both* volume and connectivity abnormalities together in ipsilateral temporal regions. A traditional group analysis is therefore insensitive to within-patient features. In contrast, hierarchical modelling uses within-patient information to investigate this relationship. Its use in this context represents one of the key novelties of our study.

**
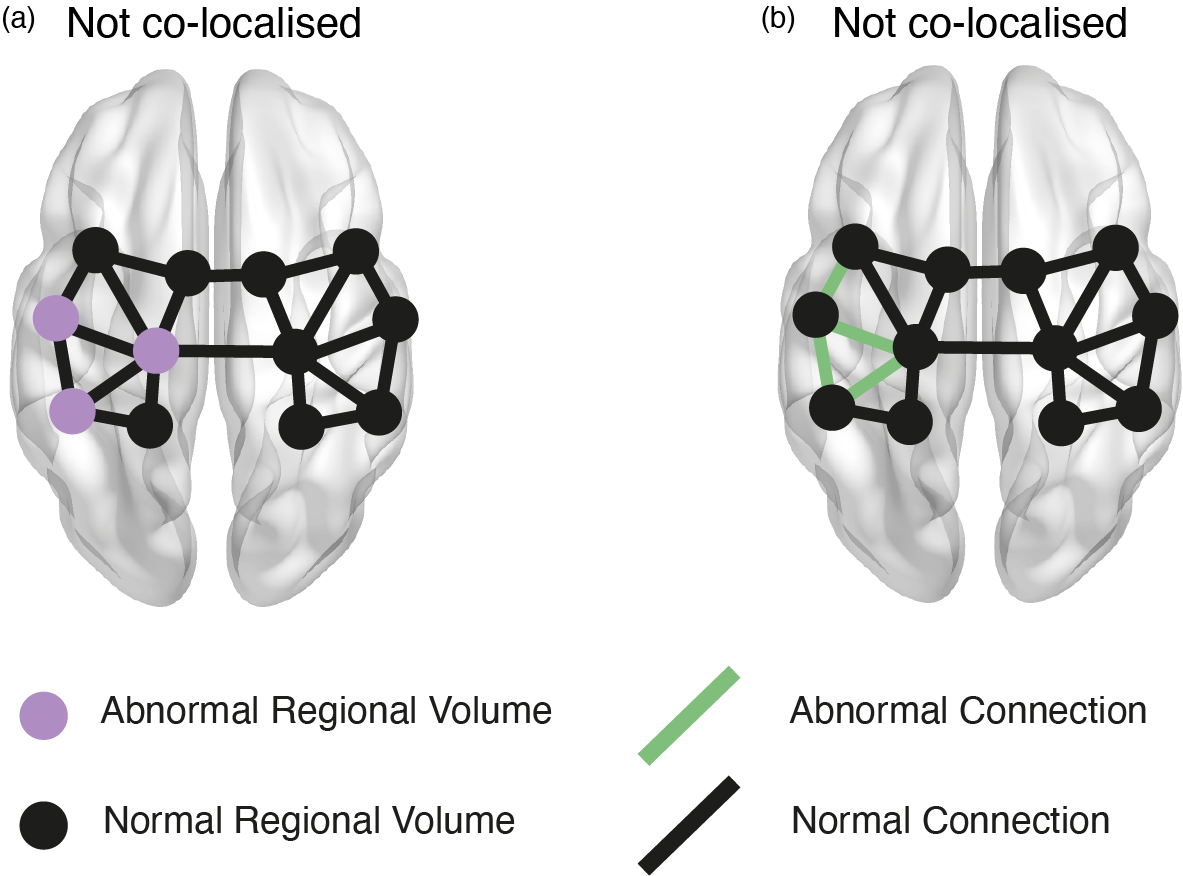
**

Figure S1: Individuals may have (a) only volumetric or (b) only connectivity abnormalities.

Additionally, patients may have both volumetrics and connectivity abnormalities but these may or may not be co-localised. This is illustrated in Figure S2.


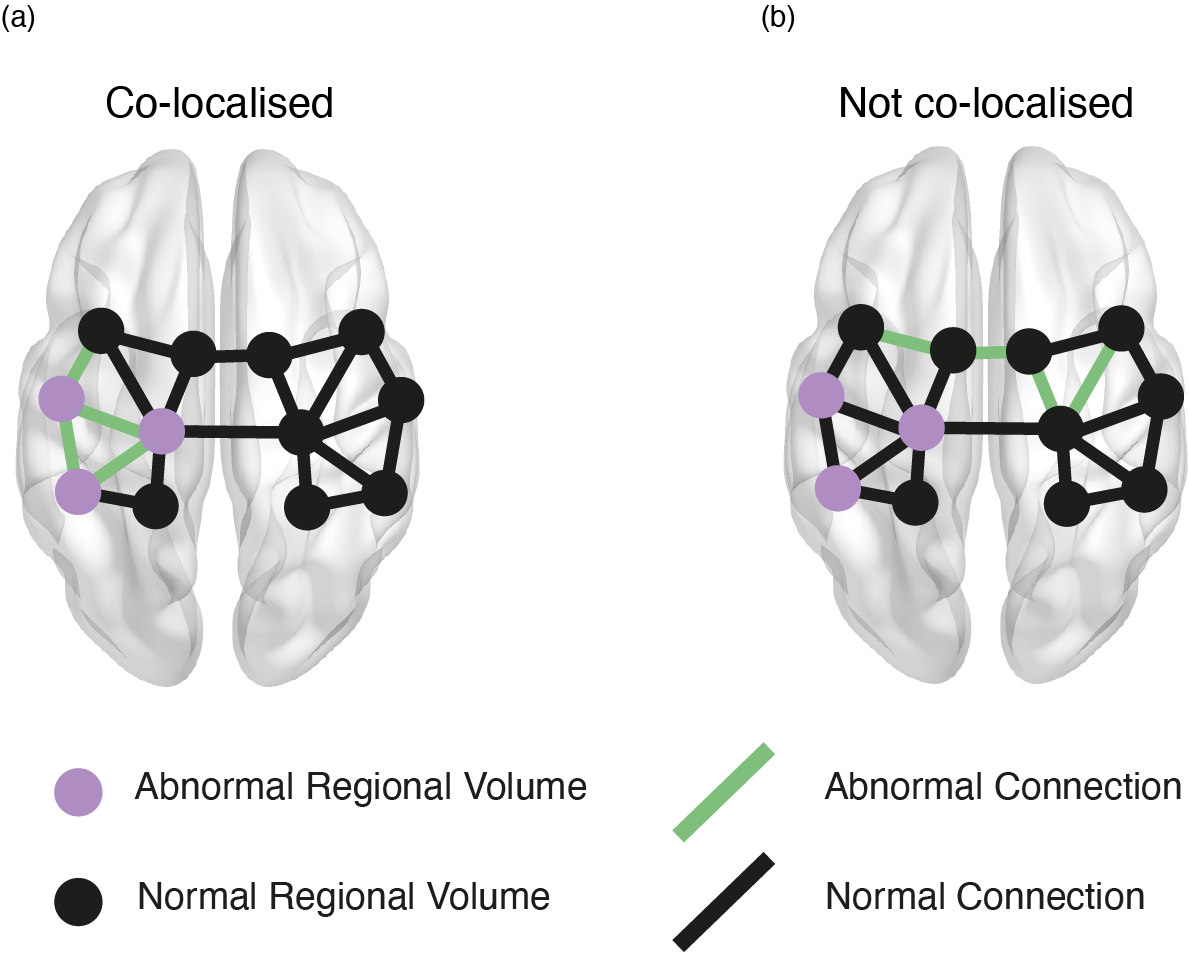


Figure S2: Individuals may have both volumetric and connectivity abnormalities but these abnormalities may (a) or may not (b) be co-localised.

**Supplementary Methods 2 - Different Analyses**

In this paper, we use different statistical methods to improve our understanding of the relationship between grey matter volumetric abnormalities and connected white matter structural connectivity abnormalities in TLE. In this section, we discuss the need for each of these different analyses.

Our group-level analysis compares where these abnormalities are on average in our cohort. Next, our hierarchical modelling approach uses within-patient abnormalities to describe the relationship between abnormalities in general, which may reflect neurobiological processes such as Wallerian degeneration. Finally, dice similarity quantifies the extent to which abnormalities co-localise within specific patients.

Using our hierarchical model, it is necessary to separately model whether volumetric abnormalities coincided with adjacent connectivity abnormalities ***and*** whether connection abnormalities coincided with adjacent volumetric abnormalities. If, for example, white matter connection abnormalities were far more widespread than grey matter volumetric abnormalities, but these abnormalities were unrelated, then connection abnormalities may still be larger in the presence of volumetric abnormalities. This is because the volumetric abnormalities could occur in a subset of the regions with connection abnormalities. However, in this scenario, it is unlikely that volume abnormalities would be significantly larger in the presence of connectivity abnormalities, since almost all abnormal connections would connect regions with normal volumes. Hence, we model both cases, i.e. whether connection abnormalities may still be larger in the presence of volumetric abnormalities **and** whether volume abnormalities would be significantly larger in the presence of connectivity abnormalities. Since our results are significant in both models, this suggests that there is a spatial relationship between volume and connectivity abnormalities.

The hierarchical approach models the relationship in individual patients and aggregates the effect across the full cohort into one estimate. Although it models the relationship in individual patients, the patients themselves are included as random effects in the model. This aggregation across patients means that it is difficult to interpret the effect within specific patients of interest. Clinically, it may be useful to understand co-localisation in individual patients because it may have an influence on the treatment choice and success. The Dice similarity coefficient is an alternative approach that quantifies the extent to which the abnormalities co-localised in individual patients.

**Supplementary Analysis 1 - Repeatability Across Parcellation Schemes: Connections Between Abnormal Volumes**

**Supplementary Analysis 1.1 - DK atlas (128 regions)**

Using the finer-grain denomination of the Desikan-Killiany atlas, we obtained similar results:

Connections between one normal and one atrophied region (estimate = -0.061 ± 0.023; p=0.008), and connections between two atrophied regions (-0.097 ± 0.043; p=0.025) had significantly reduced FA compared to connections between two normal regions. This is shown below in Figure S3. Connections between two normal regions still exhibited reduced FA in comparison to healthy controls (-0.34 ± 0.058; p<0.001).

**Supplementary Analysis 1.2 - DK atlas (233 regions)**

Connections between one normal and one atrophied region (estimate = -0.061 ± 0.019; p=0.002); and connections between two atrophied regions (-0.121 ± 0.037; p=0.002) had significantly reduced FA compared to connections between two normal regions. This is shown below in Figure S3. Connections between two normal regions still exhibited reduced FA in comparison to healthy controls (-0.305 ± 0.056; p<0.001).

**Supplementary Analysis 1.3 - DK atlas (462 regions)**

Connections between one normal and one atrophied region (estimate = -0.064 ± 0.018; p<0.001); and connections between two atrophied regions (-0.126 ± 0.032; p<0.001) had significantly reduced FA compared to connections between two normal regions. This is shown below in Figure S3. Connections between two normal regions still exhibited reduced FA in comparison to healthy controls (-0.283 ± 0.05; p<0.001).


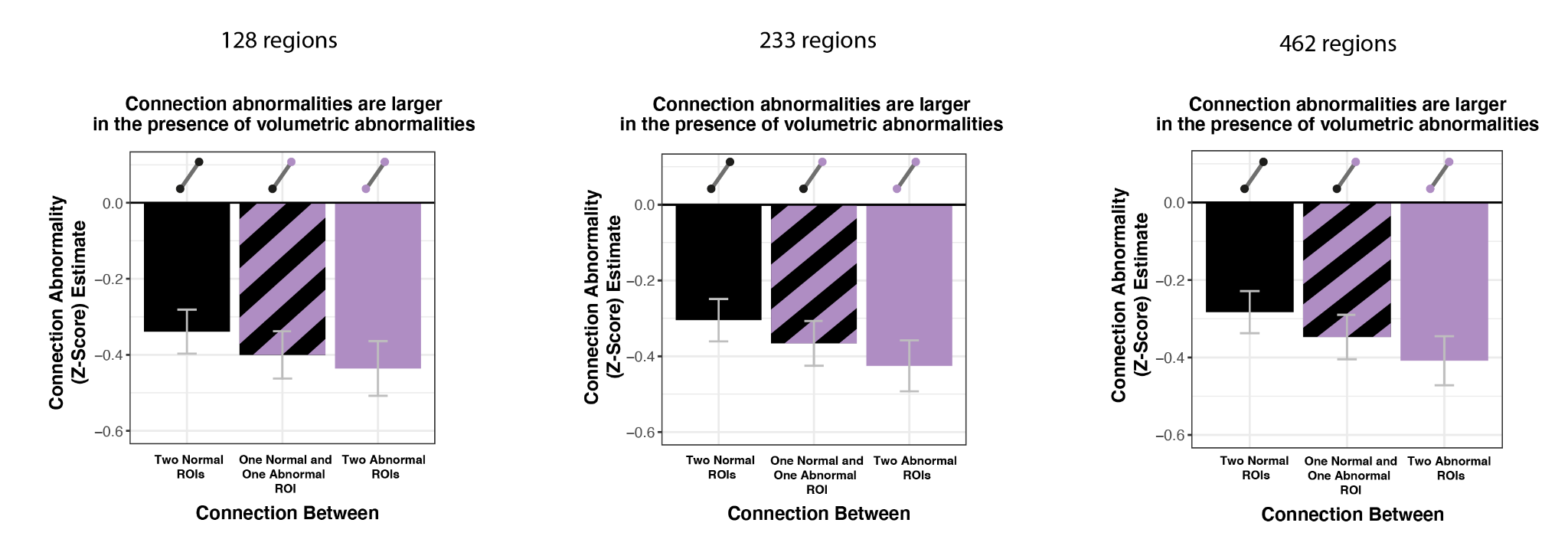


Figure S3: Hierarchical model coefficient estimates are repeatable across parcellation schemes. FA is reduced when connecting atrophied regions.

**Supplementary Analysis 2 - Repeatability Across Parcellation Schemes: Volumes Joined by Abnormal Connections**

The hierarchical model coefficient interpretations are given below for modelling volume atrophy as a function of whether connections have reduced or normal FA.

Level One:

V_ijk_ = a_k_ + b_k_C_ijk_ + 𝜀_ijk_

Level Two:

a_k_ = 𝛼_0_ + u_k_

b_k_ = 𝛽_0_ + v_k_

| Coefficient | Interpretation |
| --- | --- |
| V_ijk_ | mean volume z-score of (connected) regions i and j in subject i |
| a_k_ | mean volume z-score for regions connected by a normal connection in subject k |
| b_k_ | mean change in volume z-score for regions connected by a normal connection in subject k |
| C_ijk_ | {$\frac{1 if connection is abnormal}{0 if connection is normal}$ |
| 𝜀_ijk_ | difference between fitted and observed abnormalities for (connected) regions i and j in subject k |
| 𝛼_0_ | true mean volume z-score for regions connected by a normal connection across all subjects |
| u_k_ | difference between 𝛼_0_ and mean volume z-score for regions connected by a normal connection in subject k |
| 𝛽_0_ | true mean difference in mean volume z-score for regions connected by a abnormal connection, as compared to regions connected by a normal connection across all subjects |
| v_k_ | difference between 𝛽_0_ and mean difference in mean volume z-score for regions connected by a abnormal connection in subject k, as compared to regions connected by a normal connection in subject k |

Table S1: Hierarchical model coefficient interpretations. In all cases the volume z-score specifically denotes the mean of the two connected volume z-scores.

**Supplementary Analysis 2.1 - DK atlas (128 regions)**

Using the finer-grain denomination of the Desikan-Killiany atlas, we obtained similar results:

The volume of regions connected by abnormal connections were significantly reduced, as compared to regions connected by normal connections (estimate = -0.052 ± 0.024; p=0.031). This is shown below in Figure S4. The volumes of regions connected by normal connections still exhibited atrophy in comparison to healthy controls (-0.184 ± 0.040; p<0.001).

**Supplementary Analysis 2.2 - DK atlas (233 regions)**

The volume of regions connected by abnormal connections were significantly reduced, as compared to regions connected by normal connections (estimate = -0.049 ± 0.023; p=0.028). This is shown below in Figure S4. The volumes of regions connected by normal connections still exhibited atrophy in comparison to healthy controls (-0.161 ± 0.036, p<0.001).

**Supplementary Analysis 2.3 - DK atlas (462 regions)**

The volume of regions connected by abnormal connections were significantly reduced, as compared to regions connected by normal connections (estimate = -0.040 ± 0.019; p=0.036). This is shown below in Figure S4. The volumes of regions connected by normal connections still exhibited atrophy in comparison to healthy controls (-0.014 ± 0.031; p<0.001).


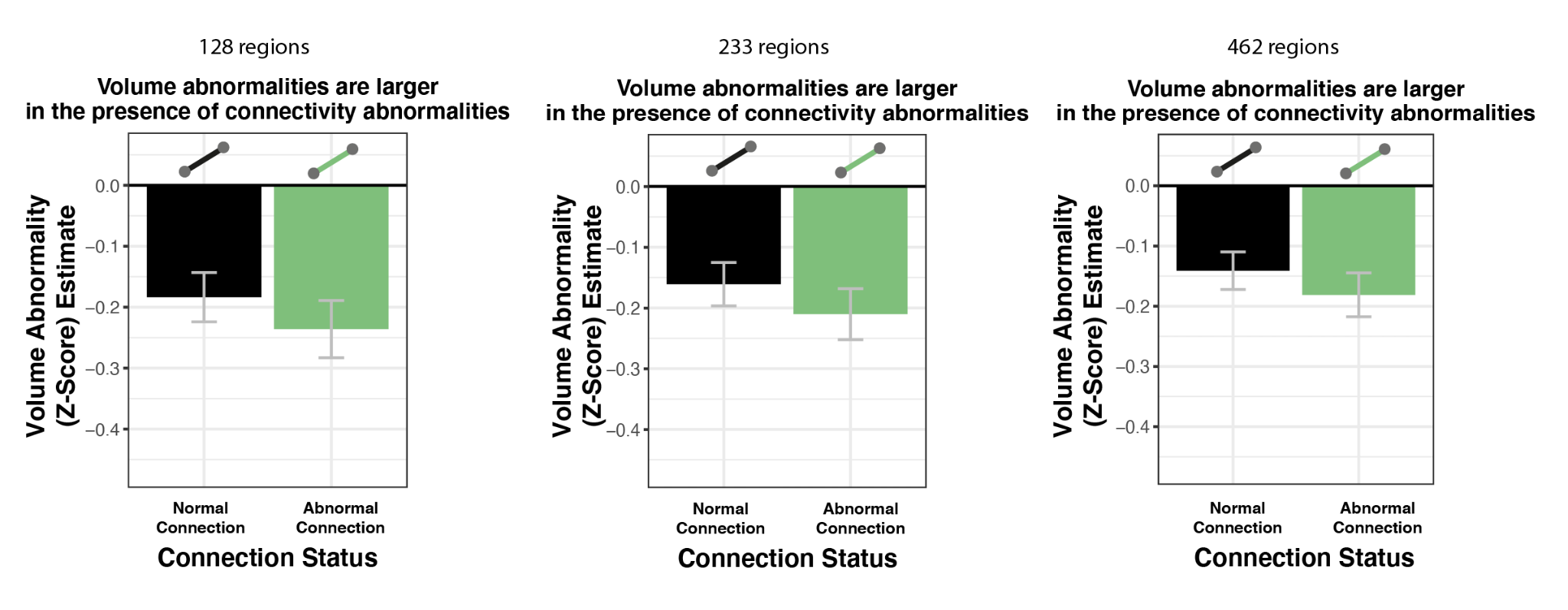


Figure S4: Hierarchical model coefficient estimates are repeatable across parcellation schemes. Volumes are reduced when connected by abnormal regions.

**Supplementary Analysis 3 - Repeatability Across Thresholds**

In the main text, abnormal connections were defined as those with FA reductions beyond a specified threshold of z = -1.96. This threshold value was scanned (1.0 to 2.5 in 0.1 steps) to ensure robustness of results. Coefficient estimates were consistent across threshold values, particularly when the threshold exceeded z=-1.5.

**Supplementary Analysis 3.1 - Repeatability Across Thresholds: Connections Between Abnormal Volumes**

**
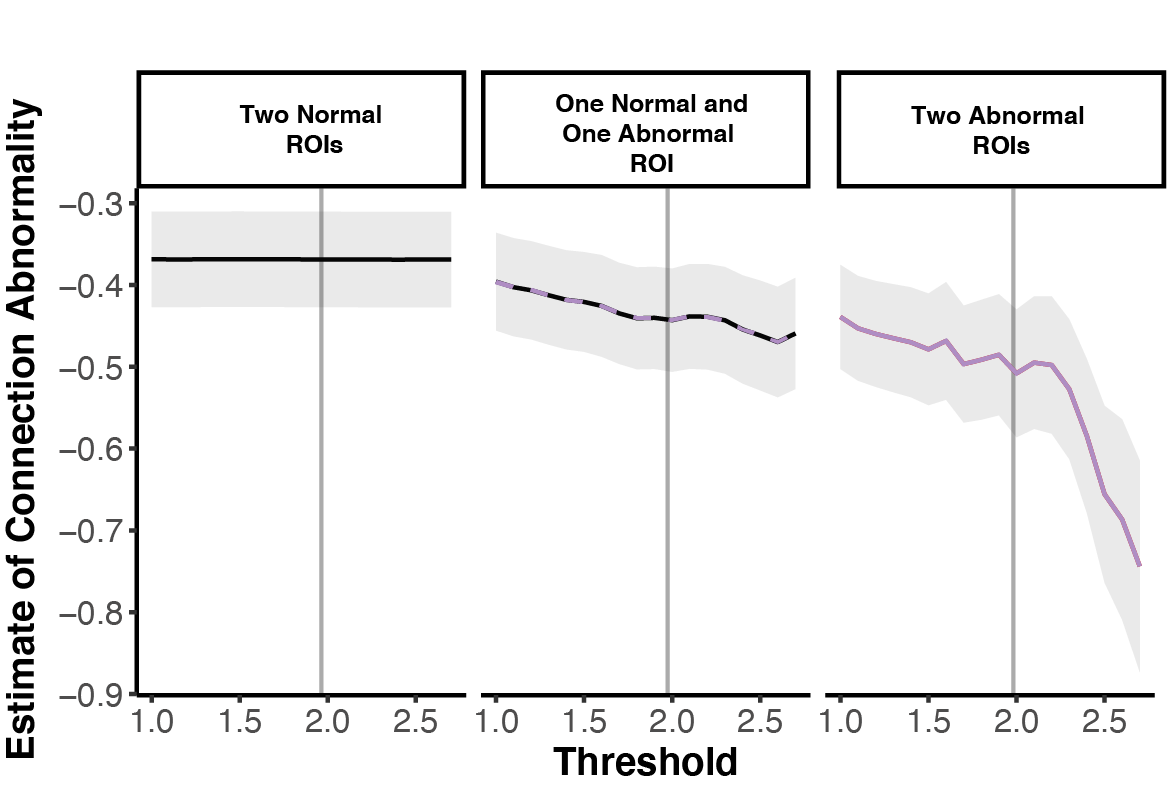
**

Figure S5: Hierarchical model coefficient estimates were repeatable across chosen threshold values. FA of connections were reduced when connected by abnormal regions. Shaded areas represent standard error of the model estimate. Vertical line indicates the threshold value reported in the main text, i.e. 1.96.

**Supplementary Analysis 3.2 - Repeatability Across Thresholds: Volumes Joined by Abnormal Connections**

**
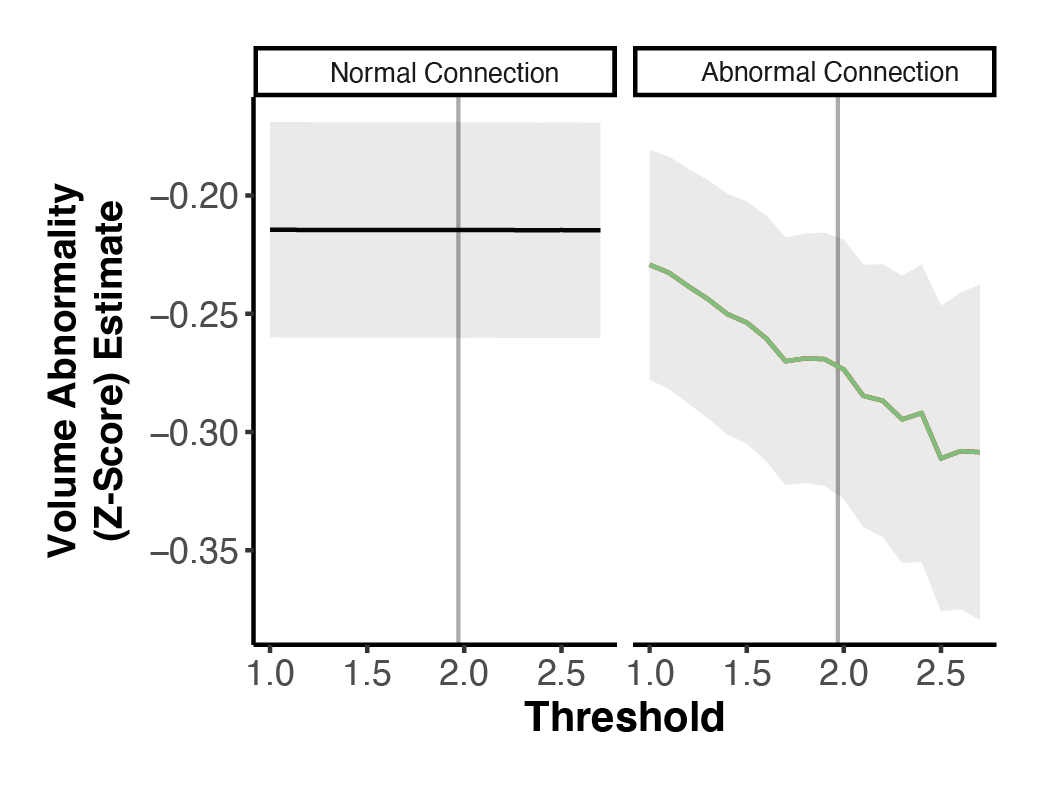
**

Figure S6: Hierarchical model coefficient estimates were repeatable across chosen threshold values. Volumes were reduced when connected by abnormal regions. Shaded areas represent standard error of the model estimate. Vertical line indicates the threshold value reported in the main text, i.e. 1.96.

**Supplementary Analysis 4 - Repeatability Across Thresholds: Colocalisation**

We repeated the colocalisation analysis with different thresholds to ensure consistency. In the main text, volumes and connections with z-scores < -1.645 were deemed abnormal, representing the bottom 5% of the data in the healthy control distribution. Figure S7 below shows the results at a lower and higher threshold. At a lower threshold of 1.5, volume abnormalities (r=0.19, p=0.03) and connection abnormalities (r=0.25, p=0.003) separately became more widespread as epilepsy duration increased, but colocalisation did not (r=-0.02, p=0.85). At a higher threshold of 1.96, there were fewer abnormalities in each patient, and as a result, more patients with no abnormalities. Using this threshold, volume abnormalities (r=0.16, p=0.09) and connection abnormalities (r=0.17, p=0.06) displayed the same trend, but did not meet significance at a 5% level.


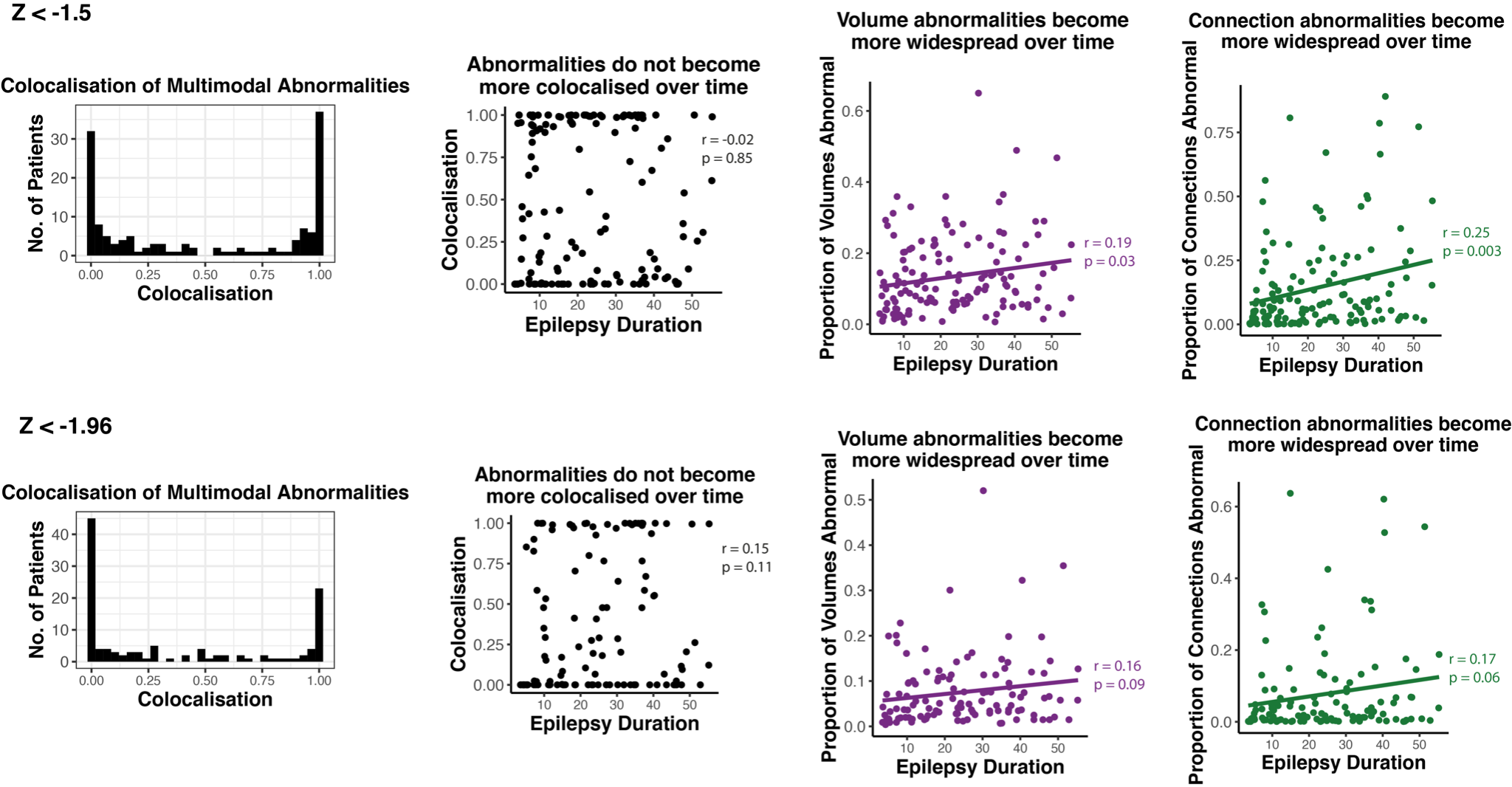


Figure S7: Colocalisation analysis at different thresholds.

**Supplementary Analysis 5 - Hierarchical Models without Ipsilateral Temporal and Subcortical Regions**

**Supplementary Analysis 5.1 - Connections Between Abnormal Volumes**

From the group-wise analysis, abnormal volumes and connections occurred primarily in ipsilateral temporal and subcortical regions. The relationship between both types of abnormality *outside of these regions* may be of interest in the context of disease manifestation and spread. As a result, we re-ran the hierarchical models excluding regions and connections contained within, or connecting to, ipsilateral temporal and subcortical regions.

Connections between one normal and one atrophied region (estimate = -0.032 ± 0.025; p=0.20), and connections between two atrophied regions (0.011 ± 0.050; p=0.83) did not have significantly reduced FA compared to connections between two normal regions. Connections between two normal regions still exhibited reduced FA in comparison to healthy controls (-0.324 ± 0.059; p<0.001).

**Supplementary Analysis 5.2 - Volumes Joined by Abnormal Connections**

The volume of regions connected by abnormal connections were not significantly reduced, as compared to regions connected by normal connections (estimate = 0.005 ± 0.025; p=0.85). The volumes of regions connected by normal connections still exhibited atrophy in comparison to healthy controls (-0.156 ± 0.045; p<0.001).

**Supplementary Analysis 6 - Hierarchical Models without Ipsilateral Temporal and Subcortical Regions by Surgical Outcome Status**

As suggested, we re-ran our analysis (hierarchical models) separately on ILAE 1 and ILAE 2+ patients. We discounted connections between regions in the ipsilateral temporal and subcortical regions, since these are typically removed in surgical resection for TLE. This allowed for comparison between a group with SOZ confirmed as being removed and a group with residual (at least partially) SOZ.

In accordance with our discussion point: “Co-localised abnormalities in multiple modalities outside of the surgically resected regions may indicate a failure to remove critical parts of epileptogenic network, leading to poor post-surgical outcomes”, we hypothesised that volumetric and connectivity abnormalities would co-localise in poor outcome patients, but not good outcome patients.

**Supplementary Analysis 6.1 - Connections Between Abnormal Volumes**

For good outcome patients, connections between one normal and one atrophied region (estimate = -0.40 ± 0.09; p=0.169), and connections between two atrophied regions (-0.38 ± 0.10; p=0.742) **did not have** significantly reduced FA compared to connections between two normal regions (estimate = -0.36 ± 0.08). However, in poor outcome patients, connections between one normal and one atrophied region (estimate = -0.48 ± 0.09; p=0.003), and connections between two atrophied regions (-0.53 ± 0.11; p=0.03) **did have** significantly reduced FA compared to connections between two normal regions (estimate = -0.38 ± 0.09). These results are displayed below (Figure S8 - Panel a).

**Supplementary Analysis 6.2 - Volumes Joined by Abnormal Connections**

Similarly, in good outcome patients, the volume of regions connected by abnormal connections **were not** significantly reduced (estimate = -0.27 ± 0.07; p=0.53), as compared to regions connected by normal connections (estimate = -0.25 ± 0.06). However, in poor outcome patients, the volume of regions connected by abnormal connections **were** significantly reduced (estimate = -0.23 ± 0.08; p=0.03), as compared to regions connected by normal connections (estimate = -0.15 ± 0.03) (Figure S8 - Panel b).


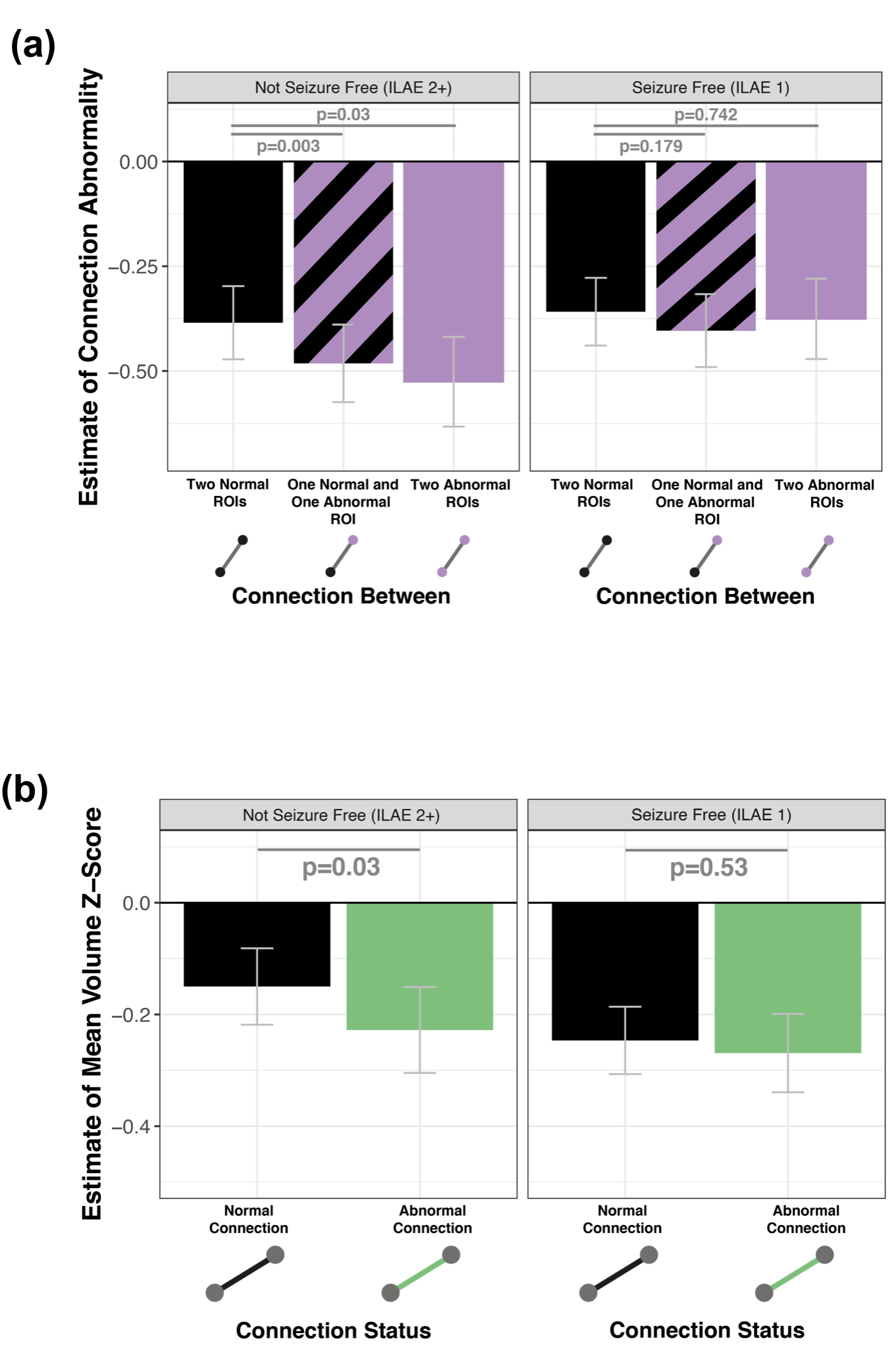


Figure S8: Hierarchical model results by surgical outcome (ILAE 1 vs ILAE 2+) excluding connections between regions in ipsilateral temporal and subcortical regions. The relationship between volumetric and connectivity abnormalities was significant in patients with a poor outcome (ILAE 2+), but not in patients with a good outcome (ILAE 1).

**Supplementary Analysis 7 - Repeatability Using Radial Diffusivity (RD) and Axial Diffusivity (AD)**

We investigated the sub-components lambda 1 (AD) and lambda 2 and 3 (RD), by re-running our analysis with the respective diffusion metric instead of FA. Since RD and AD are typically anti-correlated with FA, we hypothesised that we would see similar results, but in the opposite direction. Hatton et al. (2020) reported that patients with epilepsy typically had some increased RD and either no significant change in AD, or slight increases relative to healthy controls.

We find similar results as hypothesised or shown elsewhere. These results are detailed below separately for AD/RD.

**Supplementary Analysis 7.1: Radial Diffusivity**

At a group level, patients with TLE showed white matter connections with increased RD relative to healthy controls (Figure S9 - Panel a).

When modelled using our hierarchical approach, connections between one normal and one atrophied region (estimate = -0.60 ± 0.07; p=0.006), and connections between two atrophied regions (-0.70 ± 0.10; p=0.007) had significantly increased RD compared to connections between two normal regions (estimate = -0.52 ± 0.07) (Figure S9 - Panel b). Similarly, the volumes of regions connected by abnormal (increased RD) connections were significantly reduced (-0.27 ± 0.05; p=0.012), as compared to regions connected by normal connections (-0.20 ± 0.04) (Figure S9 - Panel c).


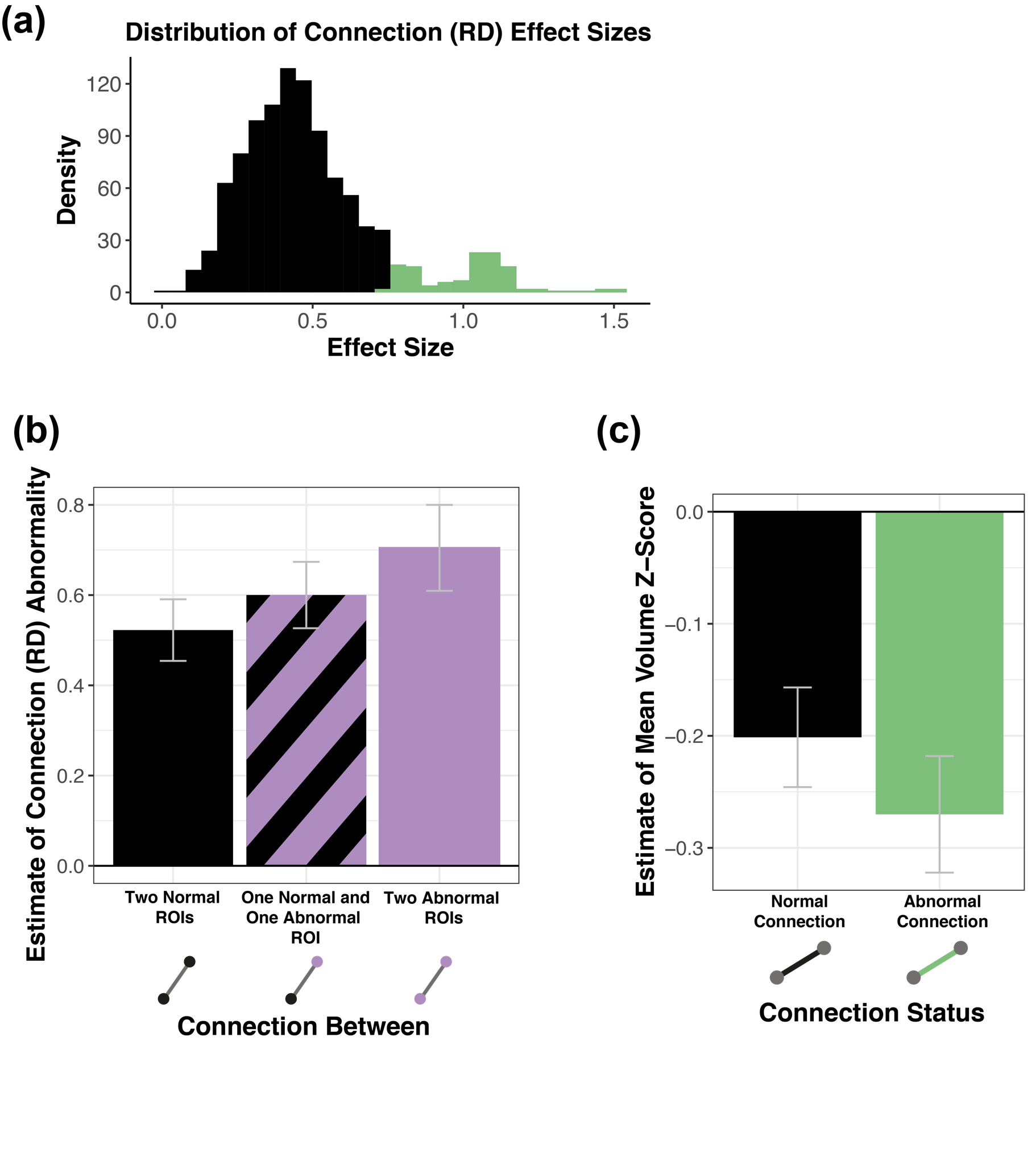


**Figure S9**: Results repeated using radial diffusivity (RD) instead of fractional anisotropy (FA). At a group-level, patients had increased RD in connections relative to healthy controls (Panel a). This increase was significantly greater in connections joining atrophied regions (Panel b). The volumes of regions was significantly reduced when connected by connections with increased RD (Panel c).

**Supplementary Analysis 7.2: Axial Diffusivity**

At a group level, patients with TLE showed some white matter connections with increased AD relative to healthy controls (Figure S10 - Panel a).

When modelled using our hierarchical approach, connections between one normal and one atrophied region (estimate = -0.57 ± 0.07; p=0.15), and connections between two atrophied regions (-0.64 ± 0.09; p=0.07) did not have significantly increased AD compared to connections between two normal regions (estimate = -0.52 ± 0.06) (Figure S10 - Panel b). Similarly, the volumes of regions connected by abnormal (increased AD) connections were not significantly reduced (-0.25 ± 0.05; p=0.09), as compared to regions connected by normal connections (-0.21 ± 0.04) (Figure S10 - Panel c).


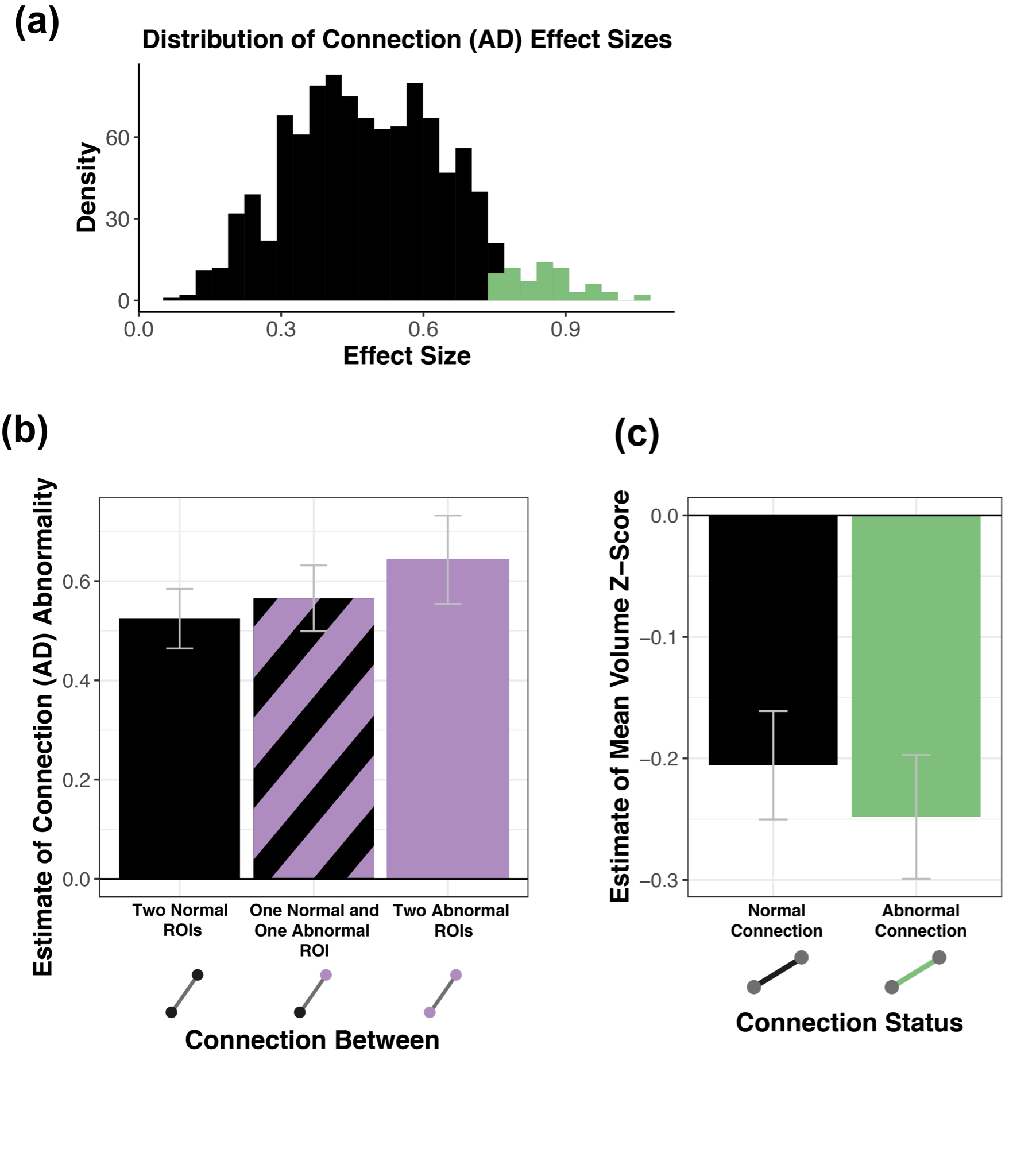


**Figure S10**: Results repeated using axial diffusivity (AD) instead of fractional anisotropy (FA). At a group-level, patients had increased AD in some connections relative to healthy controls (Panel a). This increase was greater in connections joining atrophied regions, but this was not significant (Panel b). The volumes of regions were reduced when connected by connections with increased AD, but this was not significant (Panel c).

**Supplementary Analysis 8 - Regions and Lobes**

Table S2 presents a list of the 82 regions (41 ipsilateral and 41 contralateral) used in the main analyses, and the lobe to which they were assigned.

| **Region** | **Lobe** |
| --- | --- |
| Thalamus | Subcortical |
| Caudate | Subcortical |
| Putamen | Subcortical |
| Pallidum | Subcortical |
| Hippocampus | Subcortical |
| Amygdala | Subcortical |
| Accumbens area | Subcortical |
| Banks of superior temporal sulcus | Temporal |
| Caudal anterior cingulate | Frontal |
| Caudal middle frontal | Frontal |
| Cuneus | Occipital |
| Entorhinal | Temporal |
| Fusiform | Temporal |
| Inferior parietal | Parietal |
| Inferior temporal | Temporal |
| Isthmus cingulate | Parietal |
| Lateral occipital | Occipital |
| Lateral orbitofrontal | Frontal |
| Lingual | Occipital |
| Medial orbitofrontal | Frontal |
| Middle temporal | Temporal |
| Parahippocampal | Temporal |
| Paracentral | Frontal |
| Pars opercularis | Frontal |
| Pars orbitalis | Frontal |
| Pars triangularis | Frontal |
| Pericalcarine | Occipital |
| Postcentral | Parietal |
| Posterior cingulate | Parietal |
| Precentral | Frontal |
| Precuneus | Parietal |
| Rostral anterior cingulate | Frontal |
| Rostral middle frontal | Frontal |
| Superior frontal | Frontal |
| Superior parietal | Parietal |
| Superior temporal | Temporal |
| Supramarginal | Parietal |
| Frontal pole | Frontal |
| Temporal pole | Temporal |
| Transverse temporal gyrus | Temporal |
| Insula | Frontal |

Table S2: Regions used in main analyses (D-K atlas) and associated lobes.
